# Application of Computational Studies Using Density Functional Theory (DFT) to Evaluate the Catalytic Degradation of Polystyrene

**DOI:** 10.3390/polym17070923

**Published:** 2025-03-28

**Authors:** Joaquín Alejandro Hernández Fernández, Jose Alfonso Prieto Palomo, Rodrigo Ortega-Toro

**Affiliations:** 1 Chemistry Program, Department of Natural and Exact Sciences, San Pablo Campus, Universidad de Cartagena, Cartagena de Indias D.T. y C., Cartagena 130015, Colombia; jprietop@unicartagena.edu.co; 2 Department of Natural and Exact Science, Universidad de la Costa, Barranquilla 080002, Colombia; 3 Grupo de Investigación GIA, Fundacion Universitaria Tecnologico Comfenalco, Cr 44 D N 30A, 91, Cartagena 130015, Colombia; 4 Food Packaging and Shelf-Life Research Group (FP&SL), Food Engineering Department, University of Cartagena, Cartagena 130015, Colombia; rortegap1@unicartagena.edu.co

**Keywords:** polystyrene degradation, catalytic pyrolysis, DFT, activation energy, Gibbs free energy, Gaussian 16

## Abstract

The degradation of polystyrene (PS) represents a significant challenge in plastic waste management due to its chemical stability and low biodegradability. In this study, the catalytic degradation mechanisms of PS were investigated by density functional theory (DFT)-based calculations using the hybrid functional B3LYP and the 6-311G++(d,p) basis in Gaussian 16. The influence of acidic (AlCl_3_, Fe_2_(SO_4_)_3_) and basic (CaO) catalysts was evaluated in terms of activation energy, reaction mechanisms, and degradation products. The results revealed that acid catalysts induce PS fragmentation through the formation of carbocationic intermediates, promoting the selective cleavage of C-C bonds in branched chains with bond dissociation energies (BDE) of 176.8 kJ/mol (C1-C7) and 175.2 kJ/mol (C3-C8). In contrast, basic catalysts favor β-scission by stabilizing carbanions, reducing the BDE to 151.6 kJ/mol (C2-C3) and 143.9 kJ/mol (C3-C4), which facilitates the formation of aromatic products such as styrene and benzene. Fe_2_(SO_4_)_3_ was found to significantly decrease the activation barriers to 328.12 kJ/mol, while the basic catalysts reduce the energy barriers to 136.9 kJ/mol. Gibbs free energy (ΔG) calculations confirmed the most favorable routes, providing key information for the design of optimized catalysts in PS valorization. This study highlights the usefulness of computational modeling in the optimization of plastic recycling strategies, contributing to the development of more efficient and sustainable methods.

## 1. Introduction

One of the materials that has been consolidated as fundamental for the development of modern society due to its adaptability in different sectors is plastic [1,2]. The global production of this material was about 359 million tons in 2018 [3,4], generating an increase in waste that will lead to an increasingly serious problem, such as environmental pollution and loss of natural resources [5,6]. Currently, 33 million tons of plastic waste are reported in the USA [7], while in Europe, in 2012, approximately 25 million tons were estimated, of which a worrying 38% ended up in landfills [4]. The massive accumulation of plastics poses significant risks to ecosystems and public health [8] because this material is resistant to natural decomposition [9,10], and its prolonged permanence in soils and bodies of water contributes to pollution and directly affects biodiversity [8,11]. Waste management of this material [12], including the use and/or transformation of plastics, is essential for caring for ecosystems and achieving sustainable development goals. Still, these initiatives face technical and economic challenges [13,14,15] and can generate secondary pollution [16]. Polystyrene (PS) is one of the most common plastics due to its low cost and durability. It is the fourth material produced globally [17] and is available in solid and expanded form [18]. During the recycling of PS, many of its physical properties are lost, which limits its reuse and further aggravates the problem of plastic waste [19]. Products such as food containers and disposable trays are commonly manufactured with PS, and after use, they often end up in landfills without being properly recycled [20]. The degradation of PS is a significant challenge in plastic waste management due to its recalcitrant nature, so the implementation of methods that allow its conversion into valuable products is of vital importance [21]. Pyrolysis is a technique that will enable decomposing organic materials at high temperatures in the absence of oxygen, transforming them into more useful or less harmful products [22]; however, the high temperature required to carry out the process efficiently increases operating costs and generates undesirable by-products [23,24].

A promising alternative is the use of catalysts [25,26] that improve the efficiency of the processes, favor reactions that produce chemical products with higher added value [27,28,29], and that can be reintegrated into industrial processes [30]. Among the most common catalysts used in PS pyrolysis are noble metals such as platinum (Pt) and palladium (Pd), as well as transition metals such as nickel (Ni), cobalt (Co), and iron (Fe) [31], but factors such as temperature [23], pressure, and the presence of impurities can quickly deactivate the catalyst, reducing its efficiency [32], giving rise to complex mixtures that include undesirable by-products [33]. These mixtures hinder the recovery and use of the final products, limiting the applicability of the process on a larger scale [34]. Theoretical studies on the catalytic degradation mechanism of PS at the molecular level are few [14,17,35,36]. Computational research is used to a greater extent to investigate the detailed mechanism of the pyrolysis reaction and to analyze different decomposition alternatives [37,38,39,40,41]. It also allows for simulating the chemical reactions involved in PS pyrolysis and evaluating the potential performance of different catalysts under various operating conditions [42,43], to provide a deep understanding of the chemical reactions involved and to optimize the recycling and pyrolysis processes, identifying the reaction mechanisms and the resulting products [44]. This research focused on exploring the catalytic degradation mechanisms of PS from density functional theory (DFT) simulations to predict the interactions between PS and catalysts, identify key intermediates, calculate the reaction-free energies in various routes, and understand the molecular mechanisms involved. The influence of critical parameters such as reaction temperature, catalyst nature, and PS physicochemical properties on process efficiency was analyzed.

## 2. Materials and Methods

### 2.1. Computer Simulations

DFT was combined with the hybrid Lee-Yang-Parr (B3LYP) method [45,46], which integrates three Becke parameters for the electronic structure calculations. The selection of this methodology responds to its recognized precision in describing molecular systems and catalytic surfaces, making it an appropriate tool for studying PS decomposition processes. Likewise, the 6-311G++(d,p) basis set was chosen for its capacity to accurately represent the electronic and geometric properties of the analyzed systems. This level of theory has proven particularly effective in previous studies focused on characterizing reaction mechanisms and evaluating the catalytic behavior of various materials. To ensure the reliability of the optimized geometries, frequency calculations were performed to compute the Hessian matrix, verifying that all stationary points correspond to either true minima (with no imaginary frequencies) or transition states (with a single imaginary frequency). This approach ensured the correct characterization of intermediates and transition states along the reaction pathways. Additionally, all reported energy values correspond to electronic energies calculated at 0 K, without incorporating thermal corrections.

Gaussian 16 software [47] was used to implement these calculations computationally. This software is widely recognized in the scientific community for its efficiency in predicting electronic structures, activation energies, and reaction profiles. Combining this software with the selected theoretical methodology allowed a detailed analysis of the influence of catalysts on PS degradation, providing fundamental information for the development of catalytic materials with greater efficiency and sustainability.

### 2.2. Catalysts

Catalysts with acidic and basic properties were selected. Catalysts were chosen based on their availability, cost, thermal stability, and ability to interact effectively with polymers [48]. Aluminum chloride (AlCl_3_) (Sigma-Aldrich, St. Louis, MO, USA) and iron sulfate (Fe_2_(SO_4_)_3_) (Fisher Scientific, Hampton, NH, USA), known for their ability to protonate chemical bonds and promote the formation of carbocationic intermediates, were included [49]. On the other hand, the calcium oxide (CaO) catalyst (Alfa Aesar, Haverhill, MA, USA) was selected, which favors the formation of carbanionic intermediates by proton capture [50]. These catalysts exhibit high surface area and homogeneous distribution of active sites, making them ideal for promoting selective reactions under controlled conditions [51]. Bifunctional catalysts were included in the analysis due to their potential to act simultaneously at multiple stages in the PS degradation process, maximizing conversion and minimizing the formation of solid residues [2,52,53,54,55,56,57,58,59,60,61,62,63].

### 2.3. Acid and Base Degradation Mechanisms

We investigated the mechanisms of acidic degradation mediated by carbocations and essential degradation promoted by carbanions. We also analyzed the limiting steps and key intermediates, determining the process’s efficiency. Furthermore, we identified the most favorable routes by performing Gibbs free energy (ΔG) calculations and evaluated the energy barriers associated with each process step. The data obtained allowed us to propose a comprehensive mechanistic model describing the interactions between PS and the catalysts.

## 3. Results and Discussion

### 3.1. Mechanism of Product Formation in Alkaline and Acidic Conditions

The degradation of PS in the presence of acidic and basic catalysts follows different mechanisms that influence the stability of chemical bonds and the formation of degradation products.

#### 3.1.1. Degradation in the Presence of Acid Catalysts

Acid catalysts, such as Fe_2_(SO_4_)_3_ and AlCl_3_, promote the degradation of PS by a mechanism based on the protonation of C-C bonds. This interaction generates highly reactive carbocationic intermediates, which facilitates the cleavage of the main chain and aromatic groups. Specifically, the initial protonation weakens the bonds in the side chains, promoting site-specific cleavage. Gibbs free energy calculations indicate that this mechanism has a relatively low activation barrier compared to other processes, which favors the generation of products such as styrene, toluene, and indene. However, the process can generate a mixture of side products, which may limit its selectivity and efficiency under certain operating conditions.

#### 3.1.2. Degradation in the Presence of Basic Catalysts

On the other hand, the basic catalyst CaO induces the degradation of PS through a mechanism based on the formation of carbanions. In this process, the basic catalyst captures protons from the PS structure, promoting the fragmentation of the chain by β-scission. This mechanism facilitates the formation of aromatic compounds, such as benzene and alkylated derivatives, with less formation of unwanted by-products. Analysis of bond dissociation energies (BDE) shows that, in the presence of basic catalysts, the C-C bonds in the main chain have lower cleavage energy values compared to acid catalysts, suggesting higher efficiency in fragmentation of the polymeric structure.

### 3.2. Bond Dissociation Energy

**Figure 1:**
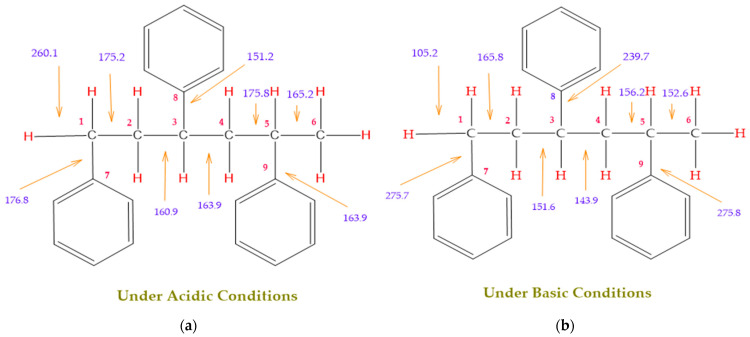
Molecular structure of the representative compound PS and cleavage energy of its predominant bonds in (**a**) acidic and (**b**) basic media (unit: kJ/mol).

Figure 1 shows the bond dissociation energies (BDEs) and how they vary for different bonds in PS under acidic conditions.

Figure 1a shows the BDE values under acidic conditions. The bonds in the branched chains, such as C1–C7, C3–C8, and C5–C9, show values of 176.8 kJ/mol, 175.2 kJ/mol, and 175.8 kJ/mol, respectively, indicating that the acidic proton attack facilitates the cleavage of these aromatic bonds. This bond weakening is due to the interaction of the proton with the phenyl groups, resulting in benzene formation. Furthermore, in the main chain, the C2–C3 (160.9 kJ/mol) and C3–C4 (163.9 kJ/mol) bonds have slightly lower values than the bonds in the branched chains, suggesting lower stability in the presence of acidic catalysts. The C1–H bond, which has a BDE value of 260.1 kJ/mol, is the strongest, reflecting its lower reactivity under acidic conditions.

In contrast, Figure 1b shows the BDE values under basic conditions. A different trend stands out: bonds in the main chain, such as C2–C3 (151.6 kJ/mol) and C3–C4 (143.9 kJ/mol), present significantly lower values than acidic conditions. This supports the alkaline catalytic mechanism, where the essential catalyst captures protons from the main chain, forming intermediate carbanions. This phenomenon facilitates β-scission reactions, generating products such as styrene, toluene, and ethylbenzene. In branched chains, bonds such as C1–C7 (275.7 kJ/mol) and C5–C9 (275.8 kJ/mol) are much more stable, indicating a lower susceptibility to cleavage. The difference in the dissociation energy of the C1–H bond is notable: under acidic conditions, the value is 260.1 kJ/mol, while under basic conditions, this bond is drastically weakened to 105.2 kJ/mol. This substantial change confirms that essential catalysts specifically affect C–H bonds, making them more susceptible to cleavage. The visual comparison between both parts of the figure shows how specific catalytic conditions (acidic or basic) selectively affect PS degradation pathways. Under acidic conditions, the aromatic (C–C) bonds in the branched chains are the main targets of the catalyst, while under basic conditions, the main chain and C–H bonds are more susceptible, allowing β-scission reactions and the formation of different chemical products.

### 3.3. HOMO-LUMO

**Figure 2:**
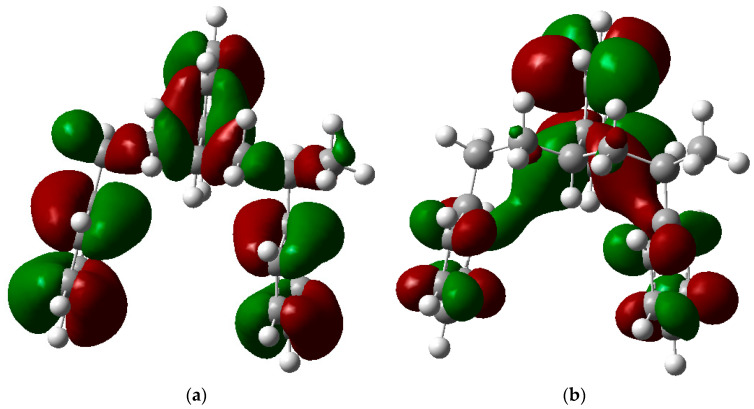
Molecular orbitals of PS (**a**) HOMO, (**b**) LUMO.

Figure 2 presents the molecular orbitals of PS, namely the HOMO and LUMO, which are fundamental for understanding the chemical reactivity of the polymer in catatolytic degradation processes. The visual representation uses two main colors: red and green, which indicate the phase of the orbitals, i.e., the regions of highest and lowest electronic density in the molecular structure.

In Figure 2a, corresponding to the HOMO, an electronic density distribution is observed in specific areas of the molecule, with well-defined lobes in red and green. These regions represent the zones where electrons are more available to participate in chemical reactions, suggesting that they are the most likely sites for interactions with catalysts. The alternation of colors indicates the symmetry and nature of the orbitals, which influences how easily electrons can be transferred. On the other hand, Figure 2b, representing the LUMO, shows a different electronic distribution, highlighting regions where electrons can be more easily accepted. The presence of the same colors (red and green) in different locations compared to the HOMO indicates a change in the orbital arrangement when the molecule transitions from one excited state to another. This is crucial in the interaction with acidic and basic catalysts, as it determines the preferred sites for polymer fragmentation. The difference in color distribution between Figure 2a,b reflects the possibility of electronic transitions and the tendency of the material to degrade under certain catalytic conditions. In this regard, acidic catalysts tend to attack the regions with higher electronic density in the HOMO (more intense red and green areas), facilitating bond cleavage. In contrast, basic catalysts promote reactions in regions where electronic density is lower in the HOMO and more prominent in the LUMO, favoring selective polymer scission mechanisms.

### 3.4. Mechanism of Product Formation Under Acidic Conditions

**Figure 3:**
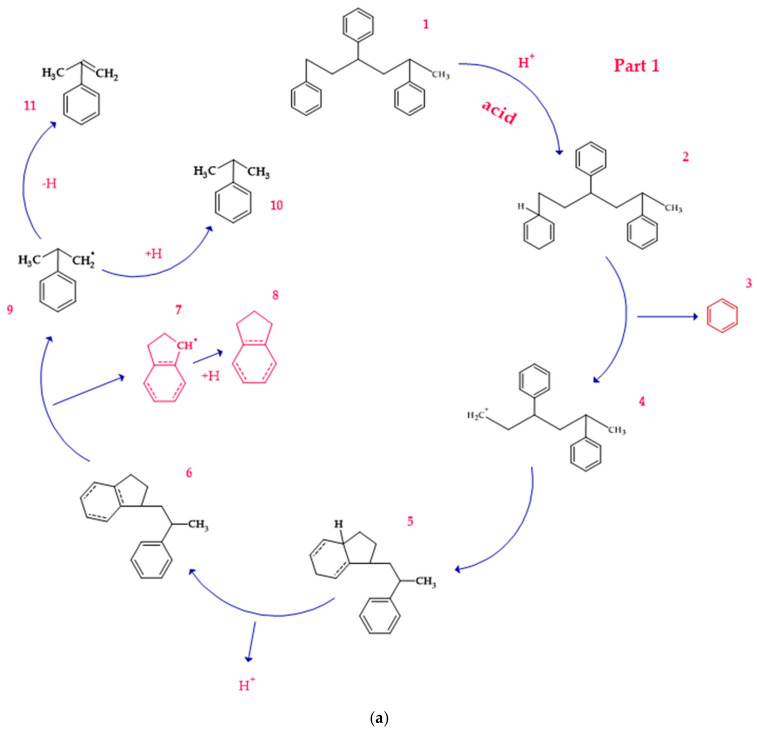

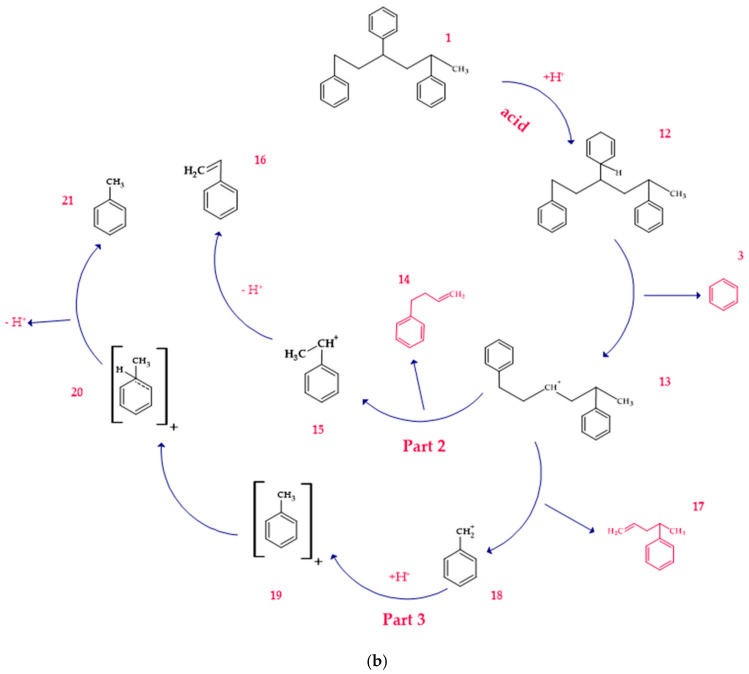
A detailed process route involves the interaction of the acid catalyst with PS. (**a**) Route 1, (**b**) Route 2 and 3.

**Figure 4:**
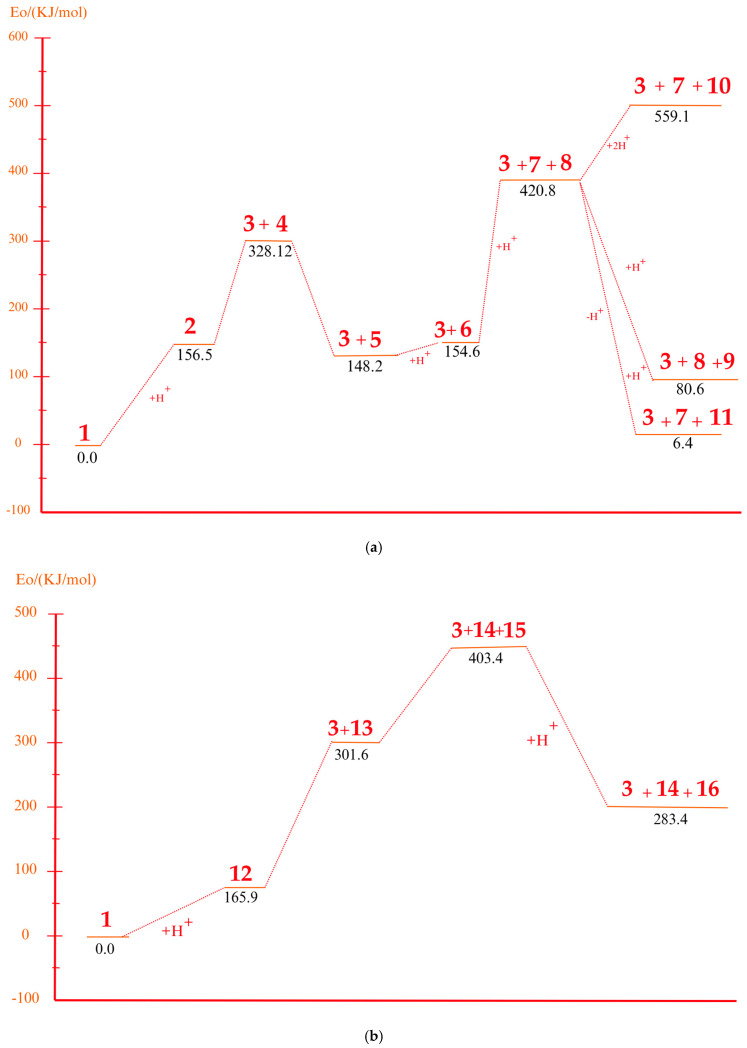

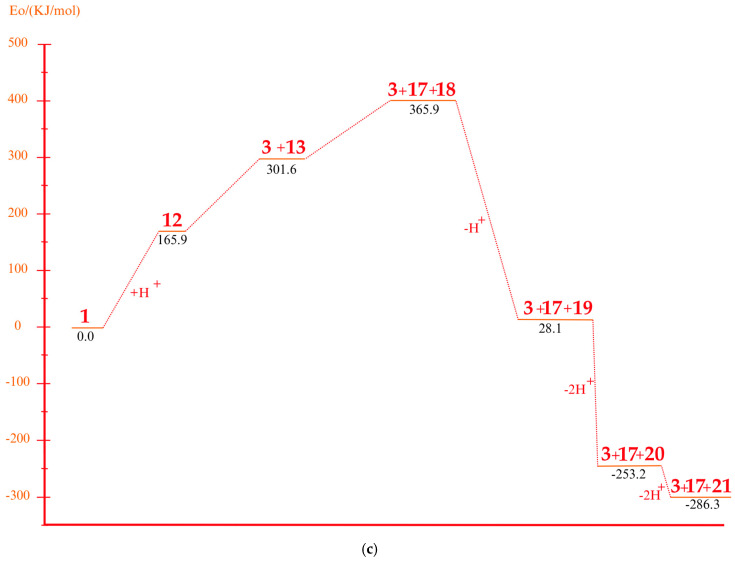
System energies are associated with the first three routes. (**a**) Route 1, (**b**) Route 2, and (**c**) Route 3.

Under acidic conditions, the catalysts facilitate the protonation of the C-C bonds of PS, generating highly reactive carbocationic intermediates [53]. These intermediates are responsible for the scission of polymer chains and the formation of aromatic compounds such as styrene, toluene, and indene [2]. Computational simulations showed that strong acid sites present in the catalysts, such as sulfate groups in Fe_2_(SO_4_)_3_, significantly reduce the activation energies, which accelerates the decomposition process. Figure 3a shows a route (part 1) identified as part of a set of five possible pathways that explain the mechanisms of product formation under acidic conditions. A detailed view of the process involving the interaction of the acid catalyst with PS is observed, which triggers a series of molecular and energetic transformations until reaching the final products. The process starts with the protonation of the phenyl group of the model compound PS, where a proton (H^+^) provided by the acid catalyst attacks the C1 carbon. This initial activation stage generates an intermediate carbocation, increasing the system’s energy by 156.5 kJ/mol concerning its initial state of 0.0 kJ/mol, as observed in Figure 4a.

Protonation weakens adjacent bonds and prepares the molecule for a controlled cleavage. Once the carbocation is formed, the C1–C7 bond of the intermediate is broken, leading to the release of benzene and the formation of a second intermediate carbocation. This cleavage process requires an additional absorption of 171.6 kJ/mol, reaching an energy maximum of 328.12 kJ/mol, one of the critical points of the reaction pathway. The release of benzene, a stable aromatic compound, facilitates the process by decreasing the structural complexity of the system. Subsequently, the intermediate carbocation undergoes a structural rearrangement, significantly releasing energy and lowering the energy state to 148.2 kJ/mol. This exothermic step temporarily stabilizes the system by redistributing the electronic charges and improving the stability of the carbocation. Proton elimination then occurs, generating a new intermediate compound with a slight increase in energy up to 154.6 kJ/mol. This moderate increase suggests proton elimination requires less energy than the previous steps.

All the optimized molecules can be seen in Table 1 together with their numbering.

In a key step of the mechanism, the C3–C4 bond is cleaved, generating two free radicals named 7 and 8. This energetically expensive bond cleavage raises the system’s energy to 420.8 kJ/mol, constituting a critical barrier in the energy profile. The free radicals formed are highly reactive species that are crucial in creating the final products. From here, the free radicals follow divergent paths. Free radical 7 captures a hydrogen atom, a process that stabilizes the system and leads to the formation of indene, releasing a considerable amount of energy until reaching a state of 80.6 kJ/mol. The formation of indene is one of the most favorable routes due to its aromatic nature, which provides excellent stability to the final product.

On the other hand, free radical 8 presents two possible routes. In the first, it undergoes the elimination of a hydrogen atom, forming α-methylstyrene. However, this process requires an energy absorption of 559.1 kJ/mol, representing the highest energy peak of the entire route and evidencing an energetically unfavorable stage. In the second route, radical 8 captures a hydrogen atom, leading to the formation of isopropylbenzene. This process results in a significant energy release, bringing the system to a final stable state of 6.4 kJ/mol, the most energetically favorable point. Routes 2 and 3 are shown in Figure 3b, where the acidic catalytic degradation of PS is observed, with new key molecular transformations supported by the energy profile shown in Figure 4b and Figure 4c, respectively. Both routes continue the detailed analysis of PS fragments under an acid catalyst’s influence, producing intermediate species and high-added-value products. The process starts similarly to Route 1, where protonation of the phenyl group activates the system and facilitates the subsequent steps. In Route 2, an intermediate carbocation is formed with an energy requirement of 165.9 kJ/mol. This value is slightly higher than that of the initial protonation observed in Route 1 (156.5 kJ/mol), suggesting a higher structural strength of the activated bond. The cleavage of the adjacent C–C bond from the carbocation occurs, leading to the formation of an intermediate compound with an energy of 301.6 kJ/mol. This step is energetically demanding, evidencing a critical barrier in the process. The resulting intermediate undergoes a series of structural rearrangements, partially stabilizing the system before forming the final products. In the next key step, the system reaches an energy peak of 403.4 kJ/mol, which is associated with generating free radicals and further fragmentation of the polymer structure. This energy barrier is considerably high and suggests that the cleavage of multiple bonds in the PS polymer chain requires a significant energy input under acidic conditions. The process then continues with hydrogen capture, leading to the formation of more stable products. The system’s energy decreases dramatically to 283.4 kJ/mol, representing the stabilization stage by forming final products such as ethylbenzene and benzene, highly stable and energetically favorable aromatic species.

### 3.5. Mechanism of Product Formation Under Alkaline Conditions

**Figure 5:**
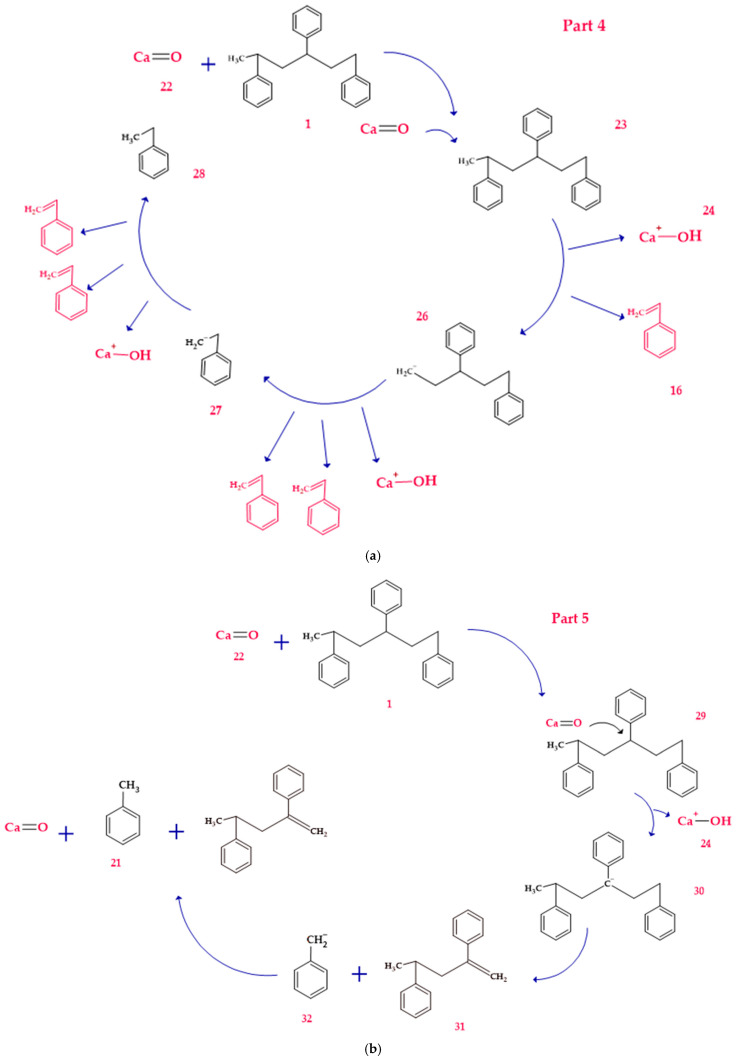
A detailed process route involves the interaction of the basic catalyst with the PS. (**a**) Route 4 and (**b**) Route 5.

On the other hand, under basic conditions, the predominant mechanism involves the deprotonation of PS chains by the CaO catalyst. This process generates carbanions that undergo β-cleavage reactions, giving rise to aliphatic and aromatic hydrocarbons [33]. DFT calculations show that the essential catalysts effectively stabilize the carbanions, reducing the energy barriers and favoring the formation of specific products such as styrene and benzene. Figure 5 shows a comprehensive mechanistic model describing the interactions between PS and the catalysts.

Route 4, as shown in Figure 5, is observed under alkaline conditions. PS interacts with an alkaline environment mediated by Ca=O and other alkaline species, generating a series of reactions that culminate in stable products such as alkylbenzenes, benzene, and hydroxylated derivatives.

The process begins with the interaction between Ca=O and the model polystyrene. In this first stage, the energy system starts at 0.0 kJ/mol and experiences an energy release of up to −36.4 kJ/mol, suggesting a favorable activation and initial stabilization of the molecule due to the interaction with the alkaline species. Subsequently, the formation of a reactive intermediate occurs when a lateral bond in the polymer structure is broken, raising the system’s energy to 9.2 kJ/mol. This value suggests slight energy absorption, which is consistent with breaking relatively weak C–C bonds and generating new intermediate species. The process continues with the partial decomposition of the intermediates, where the fragmentation of secondary bonds and the interaction with CaOH^+^ occur, which increases the energy to 70.4 kJ/mol. This stage is crucial since the intermediate species tend to rearrange themselves into more stable configurations, facilitating the formation of final products.

**Figure 6:**
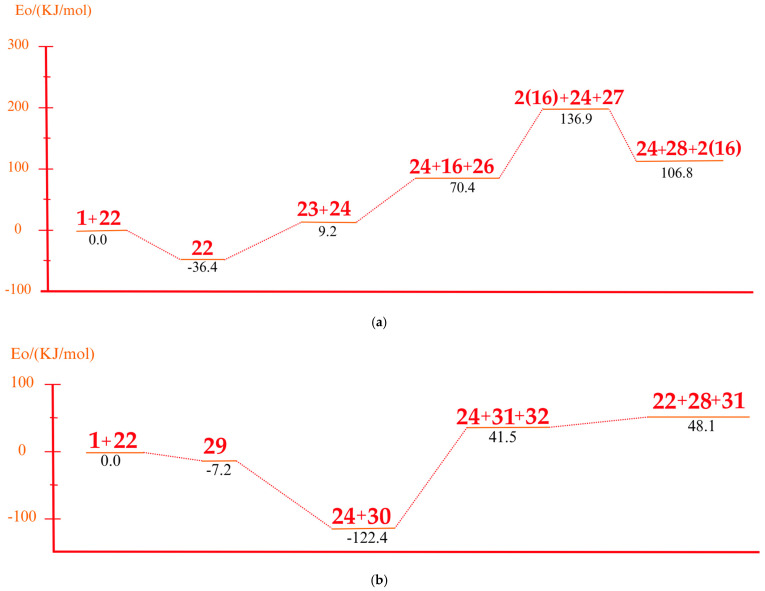
Associated system energies for the routes. (**a**) Route 4, (**b**) Route 5.

At a later stage, the highest energy peak of the process is reached, with a value of 136.9 kJ/mol. This maximum corresponds to the structural reorganization of the system and the partial stabilization of key products, such as alkylbenzenes and fragments with hydroxylated bonds. This critical energy stage reflects the resistance of the system to break more stable C–C bonds and generate reactive final species. Finally, the system experiences a significant stabilization with the release of energy up to 106.8 kJ/mol, suggesting the formation of stable final products. Figure 6a shows the associated system energies for these products, including benzene, toluene, and alkylbenzene fragments, as well as hydroxylated species generated in CaOH^+^. The final stabilization indicates that the alkaline route favors the formation of aromatic compounds with lower energy costs than the acidic route. Route 5 (see Figure 5b) represents an additional sequence of Ca=O-mediated reactions in which a selective fragmentation of the polymeric structure and the formation of stabilized aromatic products are observed, starting from an energy state of 0.0 kJ/mol. In the initial stage, the system stabilizes, decreasing the energy to −85.2 kJ/mol, suggesting that the interaction between Ca=O and the polymeric chain facilitates the breaking of weak bonds and the generation of stabilized intermediate species. This stage is crucial for activating the PS structure and preparing the following transformations. Subsequently, a rupture of secondary bonds occurs, with an energy increase up to −45.2 kJ/mol. At this point, alkyl and carbocationic intermediate species are formed and stabilized by CaOH⁺, a product of the interaction with the alkaline medium. The slight energy absorption indicates the partial resistance of some C–C bonds, although the alkaline base favors their progressive fragmentation. As the process advances, the system reaches an energy peak of 83.4 kJ/mol, corresponding to the structural reorganization of the intermediates. This critical stage involves the formation of more stable bonds and generation of alkyl aromatic products, such as styrene and benzene derivatives with methylated or ethylated groups, which are observed in Figure 5b. The energy absorption reflects the complexity of the structural transitions during this phase of the mechanism. Finally, the system experiences a partial release of energy, stabilizing at 50.1 kJ/mol, as observed in Figure 6b. This stage corresponds to forming final products, such as benzene, toluene, and other alkyl compounds derived from the controlled degradation of PS.

## 4. Conclusions

This study provides a detailed characterization of the catalytic degradation mechanisms of polystyrene (PS), highlighting the influence of catalyst type on energy stability and product selectivity. Density functional theory (DFT) calculations identified degradation pathways under acidic and basic conditions, revealing significant differences in the involved mechanisms. Under acidic conditions, AlCl_3_ and Fe_2_(SO_4_)_3_ catalysts promote the formation of carbocationic intermediates, leading to polymer chain fragmentation. However, activation barrier analysis indicates that these processes exhibit high energy requirements (~328.12 kJ/mol), suggesting that such pathways may not be feasible under standard experimental conditions. Therefore, the presence of these catalysts may induce structural rearrangements in the polymer but does not necessarily guarantee effective conversion into final products without a significant reduction in the activation barrier.

On the other hand, basic catalysts such as CaO stabilize carbanionic intermediates, facilitating β-scission reactions with significantly lower activation barriers (as low as 136.9 kJ/mol). This mechanism enables selective conversion to aromatic products such as benzene, styrene, and alkylated derivatives while minimizing the formation of undesirable by-products. From a thermodynamic perspective, Gibbs free energy (ΔG) analysis supports the feasibility of certain degradation routes. However, since the calculations were performed at 0 K, a more comprehensive evaluation incorporating thermal and enthalpic effects at relevant operational temperatures is recommended. The application of methodologies that include thermal corrections would allow for a more rigorous assessment of the energetic viability of the proposed mechanisms.

The molecular orbital analysis (HOMO-LUMO) suggests that acidic catalysts target regions of high electron density in the HOMO, whereas basic catalysts stabilize reactive sites in the LUMO, providing greater control over polymer scission. This underscores the importance of selecting catalyst nature based on the desired products. Overall, these findings emphasize the usefulness of computational approaches in optimizing PS recycling strategies and developing more efficient and selective catalysts. However, future research should integrate theoretical studies with experimental validation to confirm the feasibility of the proposed mechanisms and refine the design of catalytic strategies for PS valorization.

## Figures and Tables

**Table 1:** Optimized numbering and structure of molecules.

Optimized Structure	Number	Optimized Structure	Number
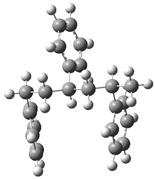	1	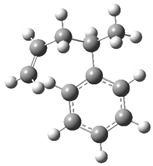	17
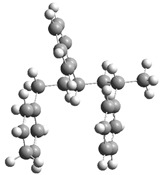	2	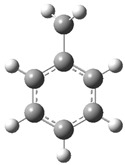	18
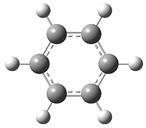	3	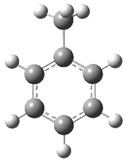	19
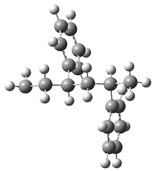	4	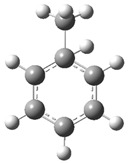	20
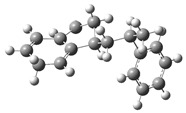	5	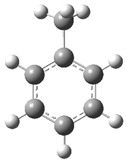	21
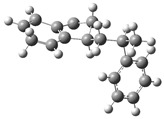	6	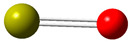	22
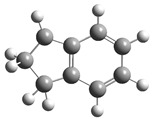	7	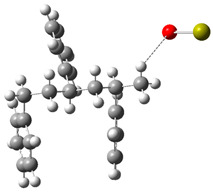	23
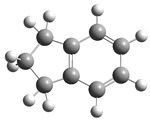	8	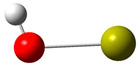	24
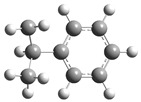	9	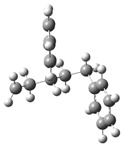	25
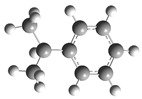	10	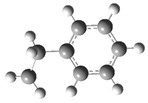	26
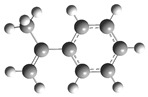	11	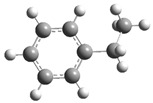	27
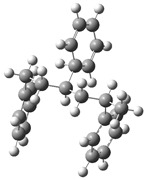	12	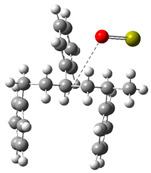	28
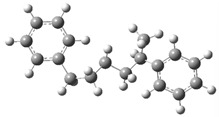	13	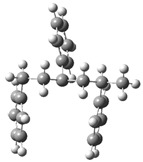	29
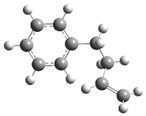	14	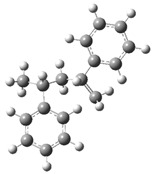	30
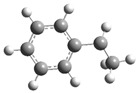	15	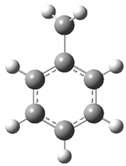	31
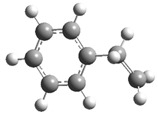	16		

## Data Availability

Data are contained within the article.
